# Oculomotor Examination of the Weapon Focus Effect: Does a Gun Automatically Engage Visual Attention?

**DOI:** 10.1371/journal.pone.0081011

**Published:** 2013-12-11

**Authors:** Heather D. Flowe, Lorraine Hope, Anne P. Hillstrom

**Affiliations:** 1 College of Medicine, Biological Sciences and Psychology, University of Leicester, Leicester, United Kingdom; 2 Department of Psychology, University of Portsmouth, Portsmouth, United Kingdom; University of Bath, United Kingdom

## Abstract

**Background:**

A person is less likely to be accurately remembered if they appear in a visual scene with a gun, a result that has been termed the *weapon focus effect* (WFE). Explanations of the WFE argue that weapons engage attention because they are unusual and/or threatening, which causes encoding deficits for the other items in the visual scene. Previous WFE research has always embedded the weapon and nonweapon objects within a larger context that provides information about an actor's intention to use the object. As such, it is currently unknown whether a gun automatically engages attention to a greater extent than other objects independent of the context in which it is presented.

**Method:**

Reflexive responding to a gun compared to other objects was examined in two experiments. Experiment 1 employed a prosaccade gap-overlap paradigm, whereby participants looked toward a peripheral target, and Experiment 2 employed an antisaccade gap-overlap paradigm, whereby participants looked away from a peripheral target. In both experiments, the peripheral target was a gun or a nonthreatening object (i.e., a tomato or pocket watch). We also controlled how unexpected the targets were and compared saccadic reaction times across types of objects.

**Results:**

A gun was not found to differentially engage attention compared to the unexpected object (i.e., a pocket watch). Some evidence was found (Experiment 2) that both the gun and the unexpected object engaged attention to a greater extent compared the expected object (i.e., a tomato).

**Conclusion:**

An image of a gun did not engage attention to a larger extent than images of other types of objects (i.e., a pocket watch or tomato). The results suggest that context may be an important determinant of WFE. The extent to which an object is threatening may depend on the larger context in which it is presented.

## Introduction

Approximately one-quarter of violent crimes in the UK and the USA involve the use of a weapon [1], [2]. Evidence suggests that victims are less likely to sustain physical injury if the perpetrator commits the crime with compared to without a weapon [3]. If injury is sustained, however, it is more likely to be lethal in crimes committed by perpetrators with weapons, with the likelihood of death being 40 times higher if the crime is committed with a gun as opposed to without a weapon [4]. As such, it is perhaps unsurprising that people's fear of dying in a gun related incident outpaces their actual risk [5].

Observing a weapon during a crime can also impact memory for the incident. Research presenting crime scenarios to laboratory participants has found that people are generally less likely to remember details about criminal perpetrators who wield weapons, a result that has been termed the *weapon focus effect* (WFE). In the seminal study that demonstrated the WFE, participants were shown a slide sequence that varied across participants in whether the criminal perpetrator was shown holding a check or a gun [6]. Results indicated that in the gun compared to the check condition, participants fixated more on the object, produced more erroneous descriptions of the perpetrator, and were less likely to identify him from a target present photo lineup. The bulk of subsequent research has confirmed WFE, finding that people are less likely to accurately remember a person when a weapon is present during encoding [7].

The psychological mechanism underlying the WFE, however, is debated in the literature. The *arousal hypothesis* was the first account put forth to explain the WFE, and it maintains that seeing a weapon causes an observer to experience arousal, which narrows the observer's focus of attention to the weapon [8], [9], [10]. As a result, attention is less likely to be directed towards visual stimuli that are peripheral to the weapon, such as the perpetrator. Consequently, observers are less likely to encode and remember information about the perpetrator's physical appearance when a weapon is present. The arousal conceptualisation of the WFE is in keeping with Easterbrook's [11] cue-utilisation model, which posits that arousal restricts the focus of attention to the most immediate or central cues in the environment. Additionally, preferential processing of threats has been demonstrated with a range of stimuli. People are faster to detect threat-related targets, such as snakes, spiders and angry faces, compared to neutral targets [12], [13], [14], [15]. [16], an effect that is known in the literature as the *threat superiority effect*. Preferential visual processing of threat-related stimuli is arguably adaptive from an evolutionary perspective. We may be biologically prepared, therefore, to fear the stimuli that posed a threat to the survival of our early ancestors (e.g., snakes and spiders). Therefore phylogenetic threat-related stimuli may have visual processing priority over ontogenetic threat-stimuli (e.g., guns, electric outlets) and neutral stimuli [17]. However, more recent work contrasting the detection of phylogenetic (e.g., spiders, snakes) and ontogenetic threat-related stimuli using a visual search paradigm has not found differences in detection speed for phylogenetic and ontogenetic threat-related stimuli, suggesting that that both ancient and recent threats are preferentially processed over nonthreatening stimuli [18], [19], [20]. Still further, perhaps the threat-value of a stimulus is not the main determinant of the speed with which a stimulus is detected; rather, the subjective relevance of a stimulus in a given context may be the driving force that predicts detection speed [21], [22].

The *unusual object hypothesis*, another account of the WFE, also focuses on context as a determinant, arguing that weapons draw attention not because they are threatening, but rather because they are unusual or unexpected in most contexts [23], [24]. In other research domains, unexpected as opposed to expected objects have been found to draw visual attention to a greater extent (e.g., [25]). With respect to the WFE, Mitchell et al. [24] tested the unusual hypothesis by presenting participants with a slide sequence in which a man removed from his briefcase nothing (i.e., control condition), a gun, or an unexpected object, which was a stick of celery. Memory performance was poorer in the celery and gun conditions relative to the control condition. The threat hypothesis would not predict these results. Thus, these findings suggest that the WFE occurs because weapons draw attention not because they are threatening but rather because in most contexts they are unexpected. Indeed, research has shown that weapons differentially engage attention depending on whether they are presented in a context in which it would be unusual to see a weapon. For example, Pickel [26] had participants view scenarios in which an observer would expect to see a weapon (i.e., a shooting range) or in a context in which observers would not expect to see a weapon (i.e., at a baseball field). Memory performance was worse when the weapon appeared in the unexpected context, thereby supporting the unusual object over the arousal hypothesis.

However, another possibility is that both the arousal hypothesis and the unusual object hypothesis are correct. In particular, although unusual objects command greater attention than expected objects, when a weapon is unexpected it might command greater attention compared to another unexpected object because of a weapon's inherently threatening nature. To illustrate, Hope and Wright [27] presented a slideshow of a simulated crime in which the target was holding a weapon, an unusual object, or a control object. While the slideshow was being presented, participants were required to monitor numbers that appeared in a corner of the screen and to press a key when an odd number appeared. Thereafter, memory for the slideshow was tested. Results indicated impaired performance for the weapon and unusual object conditions relative to the control condition. Memory impairment, however, was worse in the weapon compared to the unusual object condition, suggesting that the weapon engaged attention to a greater extent compared to the unusual object. In keeping with these results, a recent meta-analysis of the WFE literature by Fawcett and colleagues [7] found support for both the arousal and the unusual object hypotheses. Memory performance across studies was negatively impacted by weapons and unusual objects to the same extent. However, the WFE was larger in studies that employed threatening as opposed to nonthreatening scenarios. Fawcett and colleagues proposed that there are two possible interpretations of the results. First, the results could indicate that both weapon and unusual objects generate arousal. Weapons could cause arousal due to their threatening nature, whereas unusual objects cause arousal because they are surprising. Arousal, whether induced by threat or surprise, narrows attention and reduces the probability that peripheral information is encoded. Second, the results may indicate that both arousal and unusualness impact performance. In summary, extant data support the hypothesis that a weapon may engage attention either because it is viewed as threatening as well as the hypothesis that a weapon engages attention because it is unexpected.

One assumption made by all WFE theories is that weapons are drawing attention automatically. Although the results of previous studies are consistent with this stimulus-driven or bottom up depiction of attention, the results are also consistent with attention being purposefully directed to the weapons because of the goals of the observer. In a crime, safety would be a fundamental goal that might override all others, and hence, attention might be directed to the weapon to appraise the threat. Additionally, to the degree that weapons draw attention because of their unexpected nature, it can be argued that attention is top-down because of the importance of re-evaluating a situation that has not unfolded as expected (e.g., see [28]).

The first goal of the present study was to investigate whether weapons, in their own right, outside of a context of an unfolding event that may influence threat appraisal, attract visual attention to a greater extent than unusual and usual objects. The second goal was to investigate whether the effect of a weapon on attention is primarily goal-directed or stimulus-driven. If the threatening nature of weapons draws attention automatically, attention will be engaged more by weapons than unusual or usual objects. Here, we define automatic as an involuntary process. Weapons may be inherently threatening because their portrayal by the media has led them to become ontogenetically conditioned stimuli that elicit a conditioned fear response. Previous WFE research has always embedded the weapon and nonweapon objects within a larger context, which in turn could affect threat appraisals about the object. As such, we presently do not know whether weapons, when presented alone—without a larger context that would provide information regarding the intentions of an actor to use the weapon—differentially engage attention compared to nonthreatening objects. Additionally, the only studies that have measured visual behaviour in response to a weapon have used paradigms, such as visual search tasks, that cannot distinguish well between goal-directed versus involuntary attention [18], [19], [20]. What is more, the only WFE study that has examined whether weapons automatically engage attention presented the to-be-remembered target who was wielding a gun (or a book), for 30 s, and then assessed memory for the target and the object [28], [29]. Visual attention, however, was not measured at the onset of exposure to the object; hence, whether participants automatically attended to the object when it appeared could not be assessed. Additionally, the automatic vs. goal-directed nature of attentional guidance is best explored using a paradigm in which the investigator can manipulate whether attentional guidance by stimulus properties is in competition with task goals. For this reason, the present study used the gap/overlap pro/anti-saccade paradigm, which is a standard approach for measuring attentional engagement.

**Figure 1:**
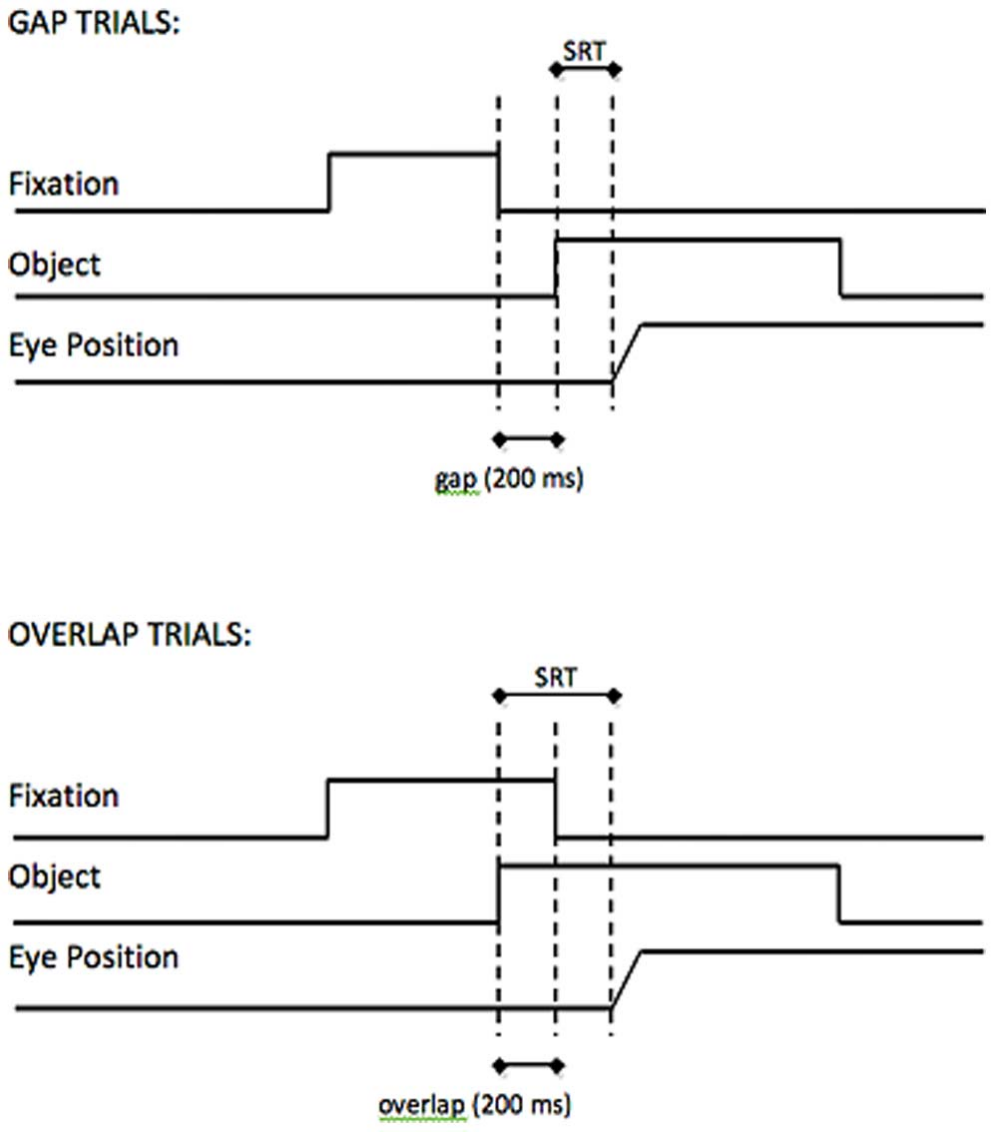
Illustration of the gap/overlap paradigm.

The gap-overlap paradigm is illustrated in Figure 1. On gap trials, there is a 200 ms time gap between the presentation of a central fixation cross and a peripheral target onscreen. On overlap trials, the fixation cross is not removed before peripheral target presentation; therefore, the fixation cross and the target overlap. Participants are instructed to either look toward (pro-saccade) or away (anti-saccade) from the peripheral target. Saccadic reaction times (SRTs) are shorter on gap compared to overlap trials. When comparing gap trials with overlap trials, the difference in SRTs occurs because of disengagement costs: namely, in the overlap condition, attention must be disengaged from the central fixation before being shifted onto the peripheral target.

In this paradigm, top-down attention is controlled by task instruction and bottom-up attention is controlled by the inherent attentional draw of the peripheral target [30]. When comparing prosaccades and antisaccade trials, prosaccades should be relatively fast because top-down and bottom-up attention both guide the eyes to the target. Antisaccades should be slower between top-down attentional guidance must overcome bottom-up attentional guidance for the participant to successfully look away from the target.

In gap trials, bottom-up attention quickly guides the eyes. In overlap trials, on the other hand, attention must be disengaged from the fixation cross before the eyes can move, so saccades are generally slower. If the bottom-up attentional pull of the target is particularly strong, that disengagement will happen faster than if the bottom-up attentional pull is weaker.

Experiment 1 employed a prosaccade gap-overlap paradigm: participants were instructed to look toward a peripheral target, which was either a weapon (i.e., a gun),an expected object nonthreatening object (i.e., a tomato), or unexpected nonthreatening object (i.e., a pocket watch). The frequency with which the target was presented was manipulated to vary how expected the object was. As the task is the same for all object types, voluntary attention should be equivalent for all. What could differ is involuntary attention. If one object type elicits more reflexive attention, then prosaccades should be quicker for those objects. Under the unexpected object hypothesis, both the gun and pocket watch objects should engage reflexive attention more than the tomato. As such, SRTs should be shorter for the gun and pocket watch object condition compared to the tomato condition. On the other hand, if a gun draws attention because of its threatening nature, then reflexive attention should be engaged more for a gun compared to both a pocket watch and tomato, as would be predicted by the arousal hypothesis.

In Experiment 2, an antisaccade paradigm was used: participants were instructed to look away from the target. In the antisaccade paradigm, reflexive attention draws the eyes to the target whereas voluntary attention directs the eyes away from the target. As in the prosaccade paradigm, voluntary attention should be equivalent for all objects, so any differences between antisaccades should reflect reflexive attention. If a gun attracts attention because of they are unexpected, then SRTs for a gun and a pocket watch objects should be longer compared to a tomoto, especially in the gap condition. On the other hand, if a gun draws attention because of its inherently threatening nature, then SRTs for a gun compared to the other objects should be longer.

## Experiment 1: Prosaccade Gap-Overlap Paradigm

### Method

#### Ethics Statement

The research protocol was reviewed and approved by the University of Leicester School of Psychology Research Ethics Committee. Written informed consent was obtained from participants directly, prior to their participation. All participants were adults over the age of 18.

#### Participants

34 students (*M* age = 19.47, *SD* = 2.85 years; n = 27 female) from the University of Leicester volunteered. All participants had normal or corrected-to-normal vision. All participants were over the age of 18.

#### Materials

Targets were black and white photographs of weapon, expected, and unexpected objects. The weapon was a handgun, the expected object was a tomato, and the unexpected object was a pocket watch. These objects were selected following a pretest, which presented participants with a number of objects and assessed whether participants could name the object and how frequently they held the object on a typical day. The aim was to select for the experiment 1) objects were approximately equally familiar across object categories, and 2) unexpected and weapon objects that were equally likely to be infrequently held by the participant. Candidate objects on the list for the unexpected category included a bowling skittle (pin), a Rubik's Twist, a life ring, a pocket watch, and a crown; the candidate weapon objects were a shotgun, a grenade, a handgun, and a knife; the candidate expected objects were common fruits and vegetables (e.g., tomato, lettuce, onion). The results of the pretesting indicated that a handgun and pocket watch were equally familiar to participants and infrequently held. The tomato was selected as the expected object because it was held more often than the other expected objects. We did not include additional exemplars within categories because so doing increased within category variability with respect to familiarity for unexpected nonthreatening objects but not for unexpected threatening objects. Namely, there was larger variability across unexpected nonthreatening objects in familiarity, because more of our participants had direct contact with these items compared to weapons. We were concerned that differences in familiarity across the unexpected threatening and nonthreatening conditions would impact attentional engagement; therefore, we elected to use one exemplar for each object category.

The tomato was 100 by 104 pixels, the handgun was 119×89 pixels, and the pocket watch was 120 by 88 pixels in size.

### Procedure

The session began by calibrating the eye tracking system, which was an Eye Link II desk mounted tracker (SR Research Ltd., Ontario) that collects 250 measurements per second. Head movements were minimized by the use of a chin rest, and thereby maintained viewing distance to 57 cm. To calibrate the eye-tracker's measurement of eye-position, the participant twice made fixations on nine sequentially appearing dots that had positions spanning the full range of the display. The eye-tracker was assumed to be calibrated successfully if the fixation positions recorded in the second sequence were within 0.70 degrees of visual angle of that predicted from measurements in the first sequence.

Participants were told to focus their eyes on the fixation cross and then saccade toward the target when it appeared. A within participants design was used to vary the experimental factors, which included object type (gun, pocket watch and tomato) and trial type (gap and overlap).

For the gap trials, the fixation cross was turned off and 200 ms later a randomly selected target was presented approximately 14 degrees from the fixation cross on the left or right side of the screen for 1500 ms. For the overlap trials, the fixation cross remained onscreen during target presentation.

There were 100 trials in total; the expected object was presented on 76 trials, the weapon on 16 trials and the unexpected object for 16 trials, with target trial order randomized. The relative frequency of the different target types was instituted to ensure that the pocket watch and gun retained their status as objects that are less frequently encountered compared to the expected object over the course of the experiment.

### Data analysis

The eye tracker measured when participants began a saccade after target onset. In order to be included in the analysis, the saccade had to begin within 0.50 degree of the fixation cross, and the SRT had to be between 80 and 500 ms following recommended practice [31]. The saccade also had to land within 7 degrees of the target [32]. For each participant, average SRT was computed by object type for the gap and the overlap conditions. Additionally, the error rate, or the rate at which participants looked away rather than toward the object, was calculated across conditions for each participant.

### Results

The error rate did not vary across object conditions. The error rate (*M* = .03) was exactly the same for each of the object conditions. As such, the error rate data were not analysed further.

The SRT data were analysed with a 2 (trial condition: gap versus overlap)×3 (object type: weapon, unexpected, expected) repeated measures ANOVA; descriptive statistics are given in Table 1. A main effect for condition was obtained, with faster SRTs in the gap compared to overlap condition, *F*(1, 33) = 33.28, *p*<.001, η_p_^2^ = .50. A main effect was also obtained for object type (weapon *M* = 339.46, unexpected *M* = 331.84, expected *M* = 340.51), *F*(2, 66) = 4.54, *p*<.05, η_p_^2^ = .12. SRTs were faster in the unexpected object condition (*M* = 331.84) compared to the expected condition (*M* = 340.51), t(33) = 3.66, p<.001.The difference in SRTs between the unexpected object and weapon conditions was not statistically significant, *t*(33) = 1.93, *p* = .06, two-tailed. The interaction between condition and object type was not significant (F = .04), indicating that the size of the gap effect did not vary in relation to object type.

**Table 1:** Group means and standard errors (SEM) of the saccadic reaction times (SRT) by object condition (weapon, expected and control) and trial type (gap and overlap) in Experiment 2, which employed the prosaccade gap-overlap paradigm.

	Trial Condition						Overall		
	gap			overlap					
	weapon	expected	unexpected	weapon	expected	unexpected	weapon	expected	unexpected
Mean	330.81	332.07	323.98	348.13	348.95	339.71	339.46	340.51	331.84
SEM	5.57	4.86	4.88	5.04	5.04	6.28	4.73	4.69	5.06

SRTs were also examined as a function of trial number to determine whether early responses to weapons and unusual objects differed compared to later responses. This analysis was undertaken to check whether attention to unusual objects and weapons was engaged to a greater extent earlier in the trial sequence when they were the most unexpected. Results indicated that SRTs did not systematically differ across the trials, suggesting that attentional engagement to the object did not wane as the number of exposures to the object increased.

### Discussion

The aim of the experiment was to test whether a gun engaged attention faster compared to an unexpected object (i.e., a pocket watch) and expected objects (i.e., a tomato). A standard gap effect was found, whereby attention was engaged faster on gap compared to overlap trials. The results further indicated that attentional engagement was not greater for a gun compared to the expected object. Interestingly, SRTs were the fastest for the unexpected object. Thus, a gun, when presented on its own without a context, did not attract attention faster than the nonthreatening stimuli that were employed.

Experiment 2 was conducted to determine whether the tendency to look toward objects was greater for a gun compared to an unexpected object and an expected object. An antisaccade gap-overlap paradigm was employed toward this end. The target was presented on the left or right side of a computer screen, and participants were instructed to look away from the target to the target's mirror position. If a gun automatically engages attention because of its inherently threatening nature, SRTs should be longer for a gun as opposed to an unexpected object and an expected object, especially on gap trials. The antisaccade gap-overlap paradigm measures the ability to supress looking towards an object. If a gun engages attention due to its threatening nature, suppression should be more challenging if the object is a weapon compared to an unexpected nonthreatening object or expected nonthreatening object. The unexpected item hypothesis was also tested. If a gun automatically attract attention because it is unexpected, then SRTs for a gun should be longer compared to an expected object. Additionally, this second study gave us the opportunity to again compare SRTs across the gun and unexpected object, as the difference between these objects approached statistical significance in Study 1.

## Experiment 2: Antisaccade Gap-Overlap Paradigm

### Method

#### Ethics Statement

The research protocol was reviewed and approved by the University of Leicester School of Psychology Research Ethics Committee. Written informed consent was obtained from participants directly, prior to their participation. All participants were adults over the age of 18.

#### Participants

29 staff and students (*M* age = 27.48, *SD* = 8.07 years; n = 15 female) from the University of Leicester volunteered. All participants had normal or corrected-to-normal vision.

#### Materials, Procedure, and Data Analysis

The stimulus materials and procedure were identical to Experiment 1 except that participants were asked to look away from the target when it appeared. Data analysis proceeded in the same manner as Experiment 1.

### Results

The error rate did not vary across trial condition or object categories: The error rate for the gap condition was 20% and for the overlap condition 18%, and the error rate was 20% for the gun, 19% for the tomato object condition, and 20% for the pocket watch object condition. These rates of error are within the range expected; the typical error rate for the antisaccade gap-overlap task is 20% [33]. Error trials were excluded from the SRT data analysis.

The SRT data were entered into a 3 (object type: weapon, unexpected, expected)×2 (condition: gap or overlap) repeated measures ANOVA. Descriptive statistics are shown in Table 2. A significant main effect was found for condition, with slower SRTs found for the overlap compared to gap condition (*M* = 381.85 versus *M* = 365.03), *F*(1,28) = 8.79, *p*<.01, η_p_^2^ = .24. Additionally, SRTs significantly varied depending on object type, *F*(2,56) = 3.43, *p*<.01, η_p_^2^ = .11. SRTs for the gun and pocket watch did not differ on average, and the means were nearly identical (*M* = 377.29 versus *M* = 377.59 ms). SRTs were shorter for the tomato when compared to each of the other object conditions (tomato *M* = 365.43 ms versus gun *M* = 377.29 ms, *t*(28) = 3.52, *p*<.01; and tomato *M* = 365.43 ms versus pocket watch *M* = 377.59 ms: *t(28) = 2.11, p<.05*.) The size of the gap effect did not vary across object type, as the interaction between condition and object type was not statistically significant (*F* = .12).

**Table 2:** Group means and standard errors of the saccadic reaction times (SRT) by object condition (weapon, expected and control) and trial type (gap and overlap) in Experiment 2, which employed the antisaccade gap-overlap paradigm.

	Trial Type						Overall		
	Gap			overlap					
	weapon	expected	Unexpected	weapon	expected	unexpected	weapon	expected	unexpected
Mean	383.84	367.25	376.71	402.42	383.58	398.11	377.29	365.43	377.59
SEM	9.10	7.30	9.33	11.65	6.51	10.34	7.37	5.91	7.16

SRTs were also examined as a function of trial number to determine whether early responses to weapons and unusual objects differed compared to later responses. SRTs did not systematically vary across trials.

## Discussion

Previous research designed to test the cause of the WFE has presented the weapon and control objects within a broader context. This research has varied the type of object carried by someone in a hypothetical scenario (e.g., [6]) or varied across hypothetical scenarios how expected a weapon would be across the given contexts (e.g., [23]). In both of these types of experimental designs, the context may be affecting differential reactions to the objects presented. Specifically, appraisals regarding the threat-value of a given object may require an assessment of the object in relation to the broader context in which it is presented. Additionally, the one study to date that has sought to examine attentional capture presented the perpetrator for 30 s [28]. Consequently, whether weapons immediately engage attention when presented is currently not known. Our aim in the present study was to examine whether a gun would engage attention faster compared to a nonthreatening object, controlling for the unusualness of the object, and to test whether that engagement was due to bottom-up or top-down direction of attention. According to the arousal hypothesis, a gun should automatically engage attention faster compared to a nonthreatening object. Alternatively, if both a gun and an unusual object attract attention faster than an expected object, then support would be found for the unusual object hypothesis.

To test our aim, Experiment 1 employed a prosaccade gap-overlap paradigm, testing whether saccadic response time (SRT) is faster on average for a gun compared to an unusual (i.e., unexpected) nonthreatening object and an usual (i.e., expected) nonthreatening object. The experimental set-up ensured that the usual object, which was a tomato, was relatively more expected than the unusual object, which was a pocket watch; the usual object was presented on 76% of the trials whereas the unusual object and gun were presented on only 12% of the trials. If weapons are inherently threatening, the gun should have attracted greater attention than the nonthreatening objects after controlling for unusualness. A standard gap effect was found, whereby SRTs were faster for the gap compared to overlap conditions. The size of the gap effect was not moderated by object type, however. Attention was not directed towards a gun any faster than the tomato or a pocket watch. Instead, SRTs were faster in the tomato object compared to the gun, and though not statistically significant, SRTs were faster for the pocket watch compared to the gun.

In Experiment 2, an antisaccade gap-overlap paradigm was used, whereby participants were instructed to look away from the object to its mirror position. Once again, a gap effect was found, but object type did not moderate the size of the gap effect. The results indicated longer SRTs for both the gun and the unusual object (i.e., pocket watch) compared to the usual object (i.e., tomato). SRTs did not differ for the gun compared to the unusual object, however. When presented alone, a gun was found to draw attention to the same extent as an unusual but nonthreatening object. Thus, the results disconfirm the hypothesis that weapons engage attention to a greater extent than other objects simply because they are inherently threatening.

The results of this study found no evidence that a gun attracts attention more than an unexpected or an expected object. Given the null results, our findings should be viewed cautiously and, of course, future research should be undertaken to replicate and extend the current paradigm. For instance, a single example of each kind of object was used in this study in order to maximize some characteristics previously uncontrolled in the WFE literature, but future studies should attempt to use other examples of weapons and unusual and usual objects. Although we controlled for variability of the stimuli within each type (by using only one example) and familiarity of the stimuli between types, it may be that other weapons would appear more threatening to participants than the gun we used.

Despite the tentative conclusions that can be drawn, this study nevertheless adds to the growing body of evidence demonstrating that whether a weapon or any object attracts attention depends on the larger context in which it is presented. Once embedded in a context, a weapon may engage attention because it is either unexpected or threatening [23], [24]. These findings are compatible with the view that stimuli gain priority in visual processing depending on their relevance in a given context [21], [22]. In this study, an unexpected weapon object did not draw attention to a greater extent than a nonthreatening unexpected object. Instead, an unexpected object, whether it was a weapon or a nonthreatening object, drew attention to a greater extent than an expected object. This may have occurred because attention automatically orients to novel objects in the visual environment (see [34], [35]). Future research should seek to replicate this finding and further explore the role of relevance in visual processing. Given that support has been found for both the unusual object hypothesis and the arousal hypothesis across the literature, additional work is needed to examine how threat-value and unusualness may work in concert to produce the WFE. In particular, as suggested by Fawcett and colleagues [7], both weapon and unusual objects might generate arousal, but for different reasons. Arousal, whether caused by threat (in the case of a weapon) or surprise (in the case of a weapon or an unusual object), narrows attention and reduces the probability that peripheral information is encoded. Additionally, it is also important to emphasize that the WFE seems to be context sensitive. As such, the size of the gap effect in relation to whether an object is threatening or unusual is probably also sensitive to context and top down processing effects.

At the start of this project, we hypothesized that a gun might capture attention even when presented alone without a context. Specifically, a gun may serve as a conditioned stimulus that causes arousal and captures attention. Since a gun is often paired with violence, the mere presentation of a photograph of a gun may elicit a fear response. The results of these two studies, however, were not in keeping with that hypothesis. Instead, the data suggest that people may be desensitized to digital presentations of weapons. Violent media are ubiquitous (e.g., [36]), and some researchers estimate that by the time a child is 12 years old, they will have seen more than 8,000 murders in (mostly simulated) digital formats [37]. To illustrate further, a recent content analysis of video game advertisements found that 68% of trailer and over 50% of print advertisements displayed a weapon, with the most common type of weapon being a gun [38]. Perhaps frequent media exposure to guns and other weapons has desensitized people to digital depictions of weapons, and consequently, a mere exposure to a photograph of a weapon no longer causes arousal. Nearly all of the research on the WFE measures responses to digital presentations of a weapon. Although the WFE has been found for both digital and live presentations, further work is needed to examine the effects of live weapon exposure on WFE. A weapon may be appraised, of course, as more threatening when presented in a live as opposed to digital context; therefore, the size of the WFE in remembering objects peripheral to the weapon may be larger in a live context. Further work is needed to examine these outstanding questions.
